# Association of Erector Spinae Plane Block on Quality of Postoperative Recovery in Patients Undergoing Video-Assisted Thoracoscopic Surgery: An Observational Cohort Study

**DOI:** 10.7759/cureus.100239

**Published:** 2025-12-28

**Authors:** Rita Miranda, Mariana Machado, Patrícia Lima, Nelma Maia, Alice Santos, Marta Guerra

**Affiliations:** 1 Anesthesiology and Perioperative Medicine, Centro Hospitalar Universitário de São João, Porto, PRT

**Keywords:** erector spinae plane block, quality of postoperative recovery, regional anesthesia, thoracic anesthesia, video-assisted thoracoscopic surgery

## Abstract

Introduction: Video-assisted thoracoscopic surgery (VATS) reduces surgical trauma compared to thoracotomy, but postoperative pain and recovery remain important concerns. The erector spinae plane block (ESPB) is a promising regional anesthesia technique, yet its impact on patient-centered outcomes, such as quality of recovery, remains underexplored.

Methods: In this prospective observational study, 54 patients undergoing elective VATS at a tertiary center were included. Patients received either an ultrasound-guided ESPB plus the institutional protocol of general anesthesia with intercostal nerve block (Group A, n=37) or the standard protocol alone (Group B, n=17). The primary endpoint was the Quality of Recovery-15 (QoR-15) score at 24 hours postoperatively, assessed numerically and by severity classification. Secondary endpoints included intraoperative and postoperative complications, length of hospitalization (in days), and numeric pain score at 24 hours postoperatively.

Results: The mean QoR-15 score was 103.4±2.6, with no significant difference between groups (t=1.04, df=52, Cohen's d=0.31, 95% CI -0.27 - 0.88, p=0.302). However, severity classification analysis showed a significant improvement (U=225.0, Z=-1.97, r=0.27, p=0.049). In Group A, 21.6% (n=8) achieved good/excellent recovery compared to none in Group B, and fewer patients were classified as poor recovery (16.2%, n=6 vs. 29.4%, n=5). No differences were observed in intraoperative and postoperative complications, numeric pain score at 24 hours postoperatively, or length of hospitalization. Importantly, ESPB did not increase anesthesia time or perioperative risk.

Conclusion: Adding ESPB to multimodal analgesia was associated with a trend toward improved postoperative quality of recovery after VATS, with a higher proportion of patients achieving good-to-excellent QoR-15 scores. These findings suggest that ESPB may be a safe and useful adjunct to enhance recovery after VATS; however, these findings require confirmation in larger randomized studies.

## Introduction

Video-assisted thoracoscopic surgery (VATS) has increasingly replaced conventional thoracotomy in many pulmonary procedures, offering advantages such as reduced surgical trauma, lower postoperative pain, and faster recovery. However, despite being a minimally invasive technique, postoperative pain remains a significant concern after VATS, potentially compromising effective ventilation, early mobilization, and overall recovery quality [1,2].

Thoracic surgery procedures, especially lung resections, are not risk-free, and as lung carcinoma is more prevalent in the elderly, often with multiple associated comorbidities, the perioperative period becomes challenging. These patients require differentiated monitoring and surveillance in the first 24-72 hours. Postoperative care includes appropriate handling of chest tubes, aggressive pain control (multimodal analgesia and regional anesthetic techniques), nausea, and multimodal rehabilitation to avoid adverse events. The application of rapid recovery protocols, the use of minimally invasive surgery, and adequate perioperative anesthetic management help to improve the prognosis of these patients [3].

The erector spinae plane block (ESPB) is a relatively recent regional anesthesia technique that has shown promise in managing thoracic pain following pulmonary surgery. Compared to thoracic epidural analgesia and paravertebral block, it offers a favorable safety profile and technical simplicity [4,5]. Although the use of ESPB in the context of VATS is gaining interest, evidence remains limited regarding its impact on broader patient-centered outcomes, such as quality of postoperative recovery [5,6].

Quality of recovery is a multidimensional concept that includes pain control, physical and emotional well-being, and functional status. The Quality of Recovery-15 (QoR-15) questionnaire is a validated and widely used tool that enables structured assessment of postoperative recovery from the patient’s perspective [7-9]. In this study, we aimed to evaluate the association of ESPB with the quality of postoperative recovery in patients undergoing VATS by comparing outcomes between those who received the block and those who did not. Specifically, it investigated the addition of an ultrasound-guided ESPB, performed by an anesthesiologist, to a standard protocol, which consists of general anesthesia and an intercostal nerve block (ICNB) performed by the surgeon.

## Materials and methods

This was a prospective, observational cohort study conducted at a tertiary care hospital. Ethical approval was obtained from the institutional review board (protocol number: 336/2023). Informed consent was obtained from all participants. Data collection was carried out from November 2023 to April 2024 through the application of the Portuguese Version-Recovery Quality Assessment Questionnaire (QoR-15) to the patients within the first 24 hours post-procedure [9] and through consultation of electronic clinical records. We collected demographic, clinical, and surgery data.

Adult patients (≥18 years) admitted to the Post-Anesthesia Care Unit of the São João Hospital and University Center after undergoing elective video-assisted thoracoscopic surgery (VATS) lobectomy, segmentectomy, or atypical/wedge resection were eligible for inclusion, while patients were excluded if the procedure was converted to thoracotomy, if they had chronic opioid use, were unable to provide informed consent, or had cognitive impairment.

Patients were divided into two groups based on the regional anesthetic technique used. Group A received an ultrasound-guided ESPB, performed by an anesthesiologist, under general anesthesia, before the start of surgery, in addition to the standard protocol. Group B followed the standard protocol, which included general anesthesia and an ICNB, using a total of 15 mL of ropivacaine 0.375%, performed by the surgeon at the end of the procedure, through infiltration of three intercostal levels (5 mL at each level). The ESPB was performed under ultrasound guidance at the T5-T6 level, using 20 mL of ropivacaine 0.375%.

Outcome measures

Primary Outcome

The Portuguese version of the QoR-15 score at 24 hours postoperatively [9], assessed both as a numeric value and categorized by severity into three classifications: poor, moderate, and good/excellent recovery. This classification system is based on the one described by Kleif J. and Gögenur I., defining score ranges as follows: excellent (136-150), good (122-135), moderate (90-121), and poor (0-89) [10]. Permission from the copyright holders to use this classification has been requested and received.

Secondary Outcomes

Intraoperative and postoperative complications, length of hospitalization (in days), and numeric pain score at 24 hours postoperatively.

Statistical analysis

As this was a prospective observational study, the sample size was determined by the number of participants consecutively recruited who met the inclusion and exclusion criteria. Data were collected using the Questionnaire (QoR-15, Portuguese version) [9] from November 2023 to April 2024. A descriptive analysis was used to summarize the results. Normality was assessed using the Shapiro-Wilk test. Continuous variables were compared using the t-test for parametric data. The Mann-Whitney U test was used to compare ordinal and continuous variables when the data did not meet parametric assumptions. Categorical variables were analyzed using the chi-square test or Fisher's exact test. All statistical analyses were conducted using IBM Corp. Released 2024. IBM SPSS Statistics for Windows, Version 30. Armonk, NY: IBM Corp.

## Results

A total of 54 participants who submitted a VATS were included in the analysis, with a median age of 68 years; of these, 77.8% (n=42) were classified as ASA III. The ESPB was added to 37 patients (group A), while the remaining 17 patients followed the standard protocol (group B), which included ICNB performed by the surgeon during the surgery. Table 1 presents the descriptive statistics for the overall population as well as for the population stratified by groups. There were no substantial differences in variables between groups, including time of anesthesia (t=1.26, df=52, p=0.215).

**Table 1:** Assessment of descriptive variables for the study population and groups. Values are presented as N (%), mean±SD, or median (interquartile range Q1-Q3). Test Statistics: U=Mann–Whitney U statistic; Z=standardized test statistic for the Mann-Whitney test; χ²=Chi-square test; t=independent-samples t-test; df=degrees of freedom. Fisher’s Exact Test was used due to small expected cell counts (<5 in some cells). p<0.05 is considered statistically significant. Group A = ESPB (erector spinae plane block) plus the standard protocol; Group B = standard protocol. ASA Classification: American Society of Anesthesiologists Classification; BMI: Body Mass Index. *One missing value.

	Total (n=54)	Group A (n=37)	Group B (n=17)	Test Statistic	
Age (years)	68 (63-73.25)	67 (63-73.50)	70 (60-73.50)	U=278.0 Z=-0.68	p=0.496
Weight* (kilograms)	70 (60-76)	70 (60-75.75)	72 (64.5-76.5)	U=292.5 Z=-0.26	p=0.797
Height*(centimeters)	163 (159.50-170.50)	164.5 (160-173.25)	159 (157-167.50)	U=198.0 Z=-2.06	p=0.039
BMI*	25.46 (22.59-28.95)	24.96 (22.71-28.19)	26.37 (21.69-30.66)	U=268.0 Z=-0.72	p=0.469
Gender				χ²=2.08 df=1	p=0.238
Male	30 (55.6%)	23 (62.2%)	7 (41.2%)		
Female	24 (44.4%)	14 (37.8%)	10 (58.8%)		
ASA Classification				Fisher’s Exact Test	p=0.865
2	10 (18.5%)	7 (18.9%)	3 (17.6%)		
3	42 (77.8%)	29 (78.4%)	13 (76.5%)		
4	2 (3.7%)	1 (2.7%)	1 (5.9%)		
Type of Surgery				Fisher’s Exact Test	p=0.317
Lobectomy	41 (75.9%)	30 (81.1%)	11 (64.7%)		
Atypical/wedge resection	7 (13%)	3 (8.1%)	4 (23.5%)		
Segmentectomy	6 (11.1%)	4 (10.8%)	2 (11.8%)		
Maintenance agent				Fisher’s Exact Test	p=0.439
Sevoflurano	45 (83.3%)	32 (86.5%)	13 (76.5%)		
Propofol	9 (16.7%)	5 (13.5%)	4 (23.5%)		
Time of surgery (minutes)	138.5 (109-190.25)	140 (118-195.50)	130 (85.50-167.50)	U=258.0 Z=-1.05	p=0.292
Time of anesthesia (minutes)	196.63±10.15	205.22±12.11	177.94±18.27	t=1.26 df=52	p=0.215

Regarding intraoperative and postoperative complications, as shown in Table 2, there were no statistically significant differences between the groups. The overall rate of intraoperative complications was 22.2% (n=12), with surgery-related issues, such as difficult resection, being the most common cause. Other intraoperative complications had an incidence below 10%, except for adverse cardiovascular events in group B (11.8%, n=2). The incidence of postoperative complications in the study population was 40.7% (n=22). Table 2 presents the distribution of postoperative complication types between the two groups. Postoperative respiratory complications were slightly more frequent in Group A, although the difference was not statistically significant (p=0.338). Conversely, the category 'Other complications' showed a modest increase in Group B, including one case of postoperative nausea and vomiting and two cases of urinary retention, none of which were observed in Group A.

**Table 2:** Intraoperative and postoperative complications for the study population and groups. Values are presented as N (%). Test Statistics: χ²=Chi-square test; df=degrees of freedom. Fisher’s Exact Test was used due to small expected cell counts (<5 in some cells). p<0.05 is considered statistically significant. Group A=ESPB (erector spinae plane block) plus the standard protocol; Group B=standard protocol.

	Total (n=54)	Group A (n=37)	Group B (n=17)	Test Statistic	
Intraoperative Complications				Fisher’s Exact Test	p=0.485
No	42 (77.8%)	30 (81.1%)	12 (70.6%)		
Yes	12 (22.2%)	7 (18.9%)	5 (29.4%)		
Type of intraoperative complications				Fisher’s Exact Test	p=0.703
Surgical	7 (13%)	4 (10.8%)	3 (17.6%)		
Cardiovascular	4 (7.4%)	2 (5.4%)	2 (11.8%)		
Respiratory	1 (1.9%)	1 (2.7%)	0		
Postoperative Complications				χ²=0.31 df=1	p=0.767
No	32 (59.3%)	21 (56.8%)	11 (64.7%)		
Yes	22 (40.7%)	16 (43.2%)	6 (35.3%)		
Type of posoperative complications					
Respiratory				Fisher’s Exact Test	p=0.338
Respiratory_No	39 (72.2%)	25 (67.6%)	14 (82.4%)		
Respiratory_Yes	15 (27.8%)	12 (32.4%)	3 (17.6%)		
Cardiovascular				Fisher’s Exact Test	p=1.0
Cardiovascular_No	51 (94.4%)	35 (94.6%)	16 (94.1%)		
Cardiovascular_Yes	3 (5.6%)	2 (5.4%)	1 (5.9%)		
Subcutaneous emphysema				Fisher’s Exact Test	p=1.0
Subcutaneous emphysema_No	46 (85.2%)	31 (83.8%)	15 (88.2%)		
Subcutaneous emphysema_Yes	8 (14.8%)	6 (16.2%)	2 (11.8%)		
Others				Fisher’s Exact Test	p=0.071
Others_No	48 (88.9%)	35 (94.6%)	13 (76.5%)		
Others_Yes	6 (11.1%)	2 (5.4%)	4 (23.5%)		

Additionally, neither the length of hospitalization (in days) nor the 24-hour postoperative numeric pain score differed significantly between the groups, as shown in Table 3. The median length of hospitalization was five days for the total cohort. Specifically, Group A had a median hospitalization of five days (interquartile range 3-6.50), while Group B had a median of four days (interquartile range 3-7) (U=279.5, Z=-0.66, r=0.09, p=0.508). Regarding the 24-hour postoperative Numeric Pain Scale, the total cohort presented a median score of 0 (interquartile range 0-2). Both Group A and Group B also reported a median score of 0, with interquartile ranges of 0-2 and 0-3.5, respectively (U=279.5, Z=-0.74, r=0.1, p=0.461). The observed effect sizes (r) were small for these variables.

**Table 3:** Length of hospitalization, Numeric Pain Score and QoR-15 Score at 24 hours postoperative analysis. Data are presented as N (%), mean±SD, or median (interquartile range Q1-Q3). Test Statistics: U=Mann–Whitney U statistic; Z=standardized test statistic for the Mann-Whitney test; r=effect sizes (r=|Z|/√N, N=54); t=independent-samples t-test; df=degrees of freedom; Cohen’s d=(MeanA–MeanB)/standard deviation pooled with 95% confidence intervals (CI). p<0.05 is considered statistically significant. Group A=ESPB (erector spinae plane block) plus the standard protocol; Group B=standard protocol. QoR-15=Quality of recovery-15 questions.

	Total (n=54)	Group A (n=37)	Group B (n=17)	Test Statistic	
Length of hospitalization (days)	5 (3-7)	5 (3-6.50)	4 (3-7)	U=279.5 Z=-0.66 r=0.09	p=0.508
Numeric Pain Scale (24 hours postoperative)	0 (0-2)	0 (0-2)	0 (0-3.5)	U=279.5 Z=-0.74 r=0.1	p=0.461
QoR-15 Score (24 hours postoperative)	103.41±2.56	105.22±3.25	99.47±3.98	t=1.04 df=52 Cohen’s d=0.31 CI 95% (-0.27 – 0.88)	p=0.302
QoR-15 Classification (24 hours postoperative)				U=225.0 Z=-1.97 r=0.27	p=0.049
Excellent and good	8 (14.8%)	8 (21.6%)	0		
Moderate	35 (64.8%)	23 (62.2%)	12 (70.6%)		
Poor	11 (20.4%)	6 (16.2%)	5 (29.4%)		

For the QoR-15 scores within the first 24 hours postoperatively, the overall cohort had a mean score of 103.41±2.56, with similar values observed between Group A (105.22 ± 3.25) and Group B (99.47±3.98). An independent-samples t-test demonstrated no significant difference in QoR-15 scores between the two groups (t=1.04, df=52, p=0.302). Cohen's d for this comparison was 0.31, with a 95% confidence interval of -0.27 to 0.88, indicating a small effect size.

In terms of QoR-15 severity classification, 64.8% (n=35) of patients had moderate scores, 14.8% (n=8) were classified as having excellent or good recovery, and 20.4% (n=11) as having poor recovery. A statistically significant difference was observed between the groups (U=225.0, Z=-1.97, p=0.049). The effect size was small to moderate (r=0.27).

**Figure 1:**
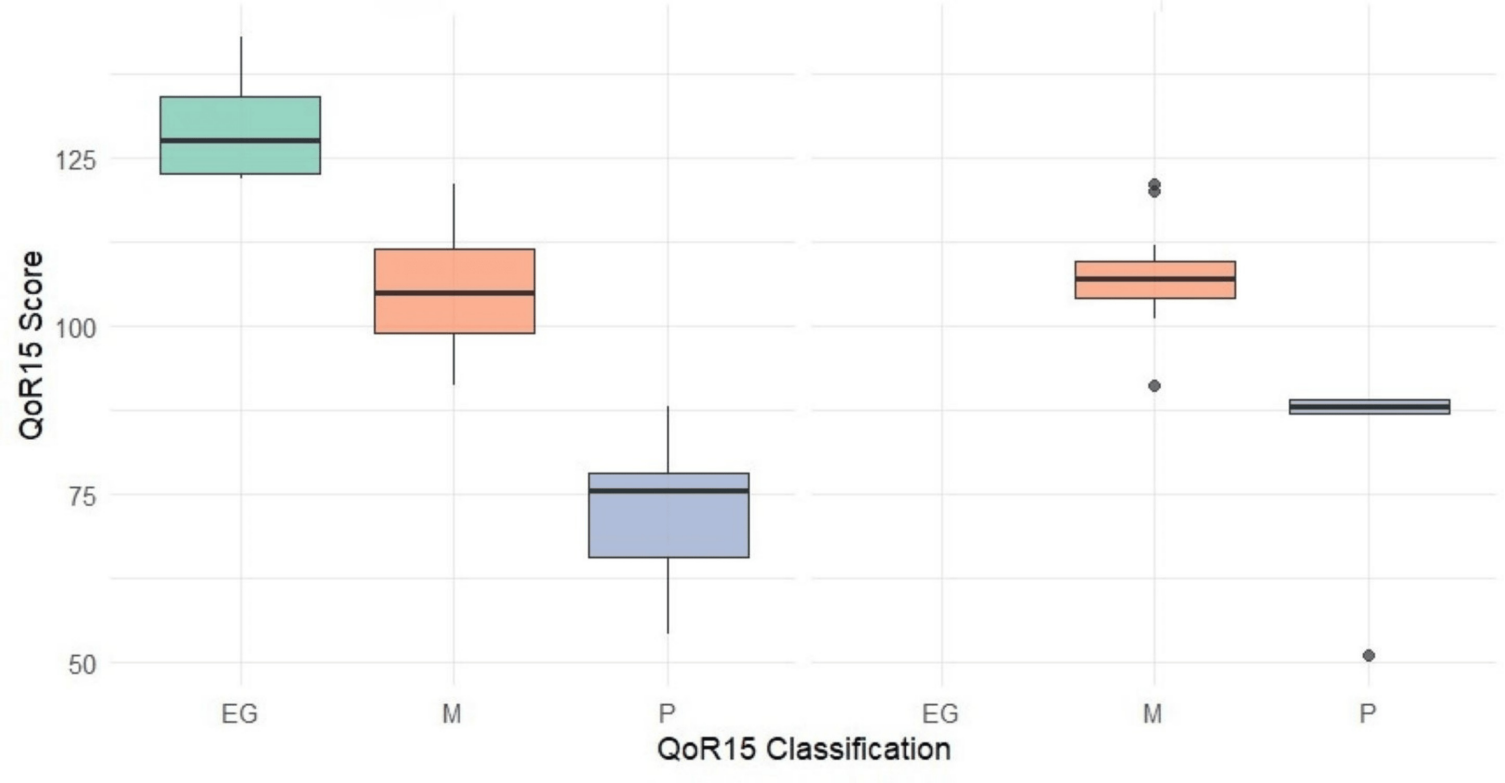
Distribution of QoR-15 score and QoR-15 classification at 24 hours postoperative by groups, group A (left) and group B (right). QoR15=Quality of Recovery-15 questions. EG=Excellent and good; M=Moderate; P=Poor.

The moderate QoR-15 category was the most frequent in both groups (62.2%, n=23 in Group A and 70.6%, n=12 in Group B). To mention that Group B had a slightly higher proportion of patients with poor QoR-15 scores (29.4%, n=5) compared to Group A (16.2%, n=6). Furthermore, no patients in Group B achieved an excellent or good QoR-15 score, in contrast to eight patients (21.6%) in Group A. These results are summarized in Table 3 and Figure 1.

## Discussion

Our data suggest that, in patients undergoing VATS, the addition of an ultrasound-guided ESPB to our institutional standard protocol was beneficial for postoperative quality recovery. The ESPB group demonstrated a statistically significant improvement in QoR-15 severity classification, with a lower percentage of patients classified as having poor recovery and a greater proportion of patients able to achieve excellent or good QoR-15 scores. Furthermore, this did not result in any substantial differences in anesthesia time.

ESPB, as a relatively novel regional anesthetic technique, presents several variations in technique and protocol performance [11-13]. Its clinical use is increasing due to its technical simplicity and favorable safety profile (especially when compared to other regional techniques such as thoracic epidural analgesia and paravertebral block). While meta-analyses have not consistently demonstrated statistically significant advantages in some outcomes over other regional techniques, growing evidence suggests that ESPB provides meaningful clinical benefits, including reduced opioid consumption and improved pain scores. This, allied to safety profile, makes ESPB a promising technique in the VATS context [5,8,14].

Recently, the evaluation of anesthetic interventions has increasingly focused on patient-centered outcomes that better capture the patient’s overall experience. Factors beyond pain and opioid consumption play a significant role in shaping patients’ perceptions of qualitative recovery after anesthesia. For this assessment, the QoR-15 questionnaire is a validated tool [7-9]. Studies in this area generally demonstrate the benefits of regional anesthetic techniques in improving postoperative QoR-15 scores [15-17]. Beyond statistical significance, they demonstrate clinically meaningful improvement, as highlighted in our study. Although there is a difference in mean QoR-15 scores at 24 hours postoperatively between groups, this was not statistically significant. However, the clinical impact is more evident in the QoR-15 severity classification, which showed a statistically significant difference.

Other factors that may influence the quality of recovery can also be explored. Postoperative lung function is a potential objective marker of recovery. Chaudhary et al. demonstrated that ESPB, compared to ICNB, resulted in improved postoperative spirometry parameters [18]. Another relevant variable is postoperative nausea and vomiting (PONV), with studies reporting a reduced incidence associated with ESPB [15,16].

In our cohort, we observed one case of PONV and two cases of urinary retention in Group B, while none were reported in Group A. Both adverse effects are commonly associated with opioid use. Although opioid consumption was not directly assessed, an increase in opioid consumption in Group B may explain this finding.

The occurrence of intraoperative complications was slightly higher in Group B (29.4%, n=5) compared to Group A (18.9%, n=7), although this difference was not statistically significant. Importantly, the addition of ESPB to our standard protocol did not have a negative impact on the intraoperative period. Surgical complications were slightly lower in Group A (10.8%, n=4) versus Group B (17.6%, n=3), as were cardiovascular events (5.4%, n=2 vs. 11.8%, n=2, respectively). Similarly, Finnerty et al. found no statistically significant differences in hemodynamic variables following ESPB [16].

Since VATS is classified as an intermediate-risk surgery [19], postoperative complications are relatively common. A propensity-matched analysis from the European Society of Thoracic Surgeons database reported a complication rate of 29.1% in patients undergoing VATS lobectomy for lung cancer [20]. In our study, the overall complication rate was 40.7% (n=22), and no significant differences were observed between the groups.

Other secondary outcomes included length of hospitalization and 24-hour postoperative numeric pain scores, and the differences were not statistically significant. Length of hospitalization is a relevant variable from a cost-effective perspective, and further investigation into the impact of ESPB in the context of VATS is encouraged. Given its potential to contribute to enhanced recovery protocols, ESPB may represent a valuable addition to perioperative care. A recent meta-analysis concluded that a continuous ESPB is associated with a reduced length of hospital stay following minimally invasive cardiac surgery [21].

This study has several limitations. As an observational, non-randomized study, it is subject to selection and confounding bias. The study groups were small and imbalanced (37 vs. 17 patients), which may limit statistical power and generalizability of the findings. Additionally, the absence of preoperative baseline QoR-15 scores limits the ability to accurately assess changes in recovery quality. Collecting baseline values would have provided a clearer comparison with postoperative scores, especially considering patient-related factors such as preoperative anxiety, which can influence perception of recovery. Data on perioperative analgesic consumption were also not collected, limiting a detailed evaluation of postoperative recovery. As a technical limitation, the ESPB was performed under general anesthesia, which precluded formal dermatomal assessment of its effectiveness, and some blocks may have been ineffective. Nevertheless, performing ultrasound-guided fascial blocks under general anesthesia reflects standard clinical practice, supporting the relevance of our findings to real-world settings. Finally, no adjustment for multiple comparisons was made, which may increase the risk of type I error. Despite these limitations, the study includes all eligible patients during the defined period and provides clinically meaningful insights using the validated QoR-15 instrument.

## Conclusions

Our study suggests potential benefits of incorporating ESPB into a multimodal analgesia regimen for VATS procedures, with a trend toward improved postoperative quality of recovery. While the primary analysis of continuous QoR-15 scores showed no statistically significant difference, a post-hoc categorical analysis indicated a higher proportion of patients achieving good/excellent QoR-15 classifications. These findings suggest that ESPB may be a safe and useful adjunct to enhance recovery after VATS; however, definitive conclusions require confirmation in prospective, adequately powered, randomized studies.
